# The Addition of *Hermetia illucens* to Feed: Influence on Nutritional Composition, Protein Digestion Characteristics, and Antioxidant Activity of *Acheta domesticus*

**DOI:** 10.3390/foods14071140

**Published:** 2025-03-25

**Authors:** Junkui Huang, Tinghao Yu, Binqiao Yuan, Jinhua Xiao, Dawei Huang

**Affiliations:** College of Life Sciences, Nankai University, Tianjin 300071, China; 2120231459@mail.nankai.edu.cn (J.H.); 1120220610@mail.nankai.edu.cn (T.Y.); 1120220611@mail.nankai.edu.cn (B.Y.)

**Keywords:** *Acheta domesticus*, nutritional characteristics, in vitro digestion, biological activity, polypeptide

## Abstract

As sales have increased in recent years, enhancing production processes and quality has emerged as a significant challenge for the cricket industry. In this study, we investigated the impact of supplementing feed with black soldier fly larvae (BSFL) on the yield, nutritional characteristics, and protein digestibility of *Acheta domesticus*. In addition, the bioactivity of house cricket proteins was compared. The results demonstrated that incorporating BSFL into feed improved the yield and nutritional characteristics of house cricket, such as crude protein levels and total phenolic content. Alterations in amino acid and fatty acid profiles also enhanced their nutritional value. In addition, 5% BSFL and 10% BSFL were more readily digested, and the protein hydrolysate of the groups fed BSFL demonstrated stronger antioxidant activity. The findings of this study can offer valuable insights into house cricket farming, protein processing, and the development of new food products.

## 1. Introduction

As one of the most popular insects for human consumption [1], *Acheta domesticus* (house cricket) is often seen as a new food because of its high protein, fatty acid, and micronutrient contents. With the increase in public acceptance and the diversity of product types, house cricket sales have risen sharply in recent years. At present, house cricket farming is still mostly based on crop powder, leaves, vegetables, etc. [2], which results in low production and poor quality; this is a major stumbling block for the cricket industry. Thus, improving the feed efficiency and nutritional quality of crickets has become the latest concern for researchers and farmers. Previously, researchers have explored the use of taro aerial parts, cassava leaves, kale, and sweet potatoes to reduce the cost of feed [3]. There are also some related studies on improving the quality of crickets with dietary phosphorus, rapeseed cakes, and fish offal [4]. Although various methods have positive effects on their development cycle and nutrient composition, no inexpensive and high-yielding method for feeding crickets has been found.

Due to their high nutritional value and ease of propagation, insects have recently emerged as the most promising and sustainable source of animal nutrition in recent years [5,6]. As a new type of feed additive, *Hermetia illucens* (black soldier fly) larvae have shown multiple benefits, such as their short life cycles, simple farming methods, and ability to yield high-quality proteins and fatty acids, as well as minerals and vitamins [7]. Furthermore, the bioactive compounds of *H. illucens* (e.g., chitin, lauric acids, and antimicrobial peptides) exhibit antioxidant and antibacterial properties and could promote animal health and survival rates [8]. Currently, black soldier fly larvae (BSFL) are widely used in livestock, poultry, and aquatic farming. The use of BSFL meal instead of fishmeal and bean pulp can greatly reduce feeding costs and improve the biological activity of organisms, especially in the aquatic industry [9]. Despite their multiple benefits, there are few articles specifically discussing BSFL as a food resource [10]. However, utilizing black soldier fly larvae as edible insect feed has great potential for providing a sustainable source of nutrients for human food.

Following the consumption of food, proteases in the human gastrointestinal tract can promote the digestion of proteins and the production of peptides and amino acids. These bioactive peptides are absorbed through the absorptive walls of the small intestine and then enter the blood circulatory system to influence and regulate physiological processes [11]. In previous studies, cricket protein was confirmed to have higher antioxidant, anti-inflammatory, anti-hyperglycemic, and antihypertensive activity through digestive simulations [12,13]. Different feeding methods result in different house cricket protein compositions, which may affect the digestion and hydrolysis of protein by pepsin and trypsin; however, few studies have evaluated this aspect. Based on previous studies, it can be speculated that *A. domesticus* fed with BSFL may have higher protein digestibility and biological activity after digestion.

Based on the abovementioned problems, the objective of this study was to investigate the nutritional composition and protein digestion characteristics of *A. domesticus* fed with BSFL powder. The bioactivity of the protein hydrolysate of the *A. domesticus* protein was contrasted in vitro. The crude nutrients, Osborne fractionation, total amino acid content, and total fatty acid content of house crickets were determined to evaluate their nutritional composition. The in vitro protein digestion characteristics of *A. domesticus* were detected using protein digestibility and the degree of hydrolysis. Finally, the antioxidant activities of cricket protein hydrolysate were detected using a variety of methods. The findings of this study can offer valuable insights into house cricket farming, protein processing, and the development of new food products.

## 2. Materials and Methods

### 2.1. Materials

Commercial *A. domesticus* powder, cricket eggs, and *H. illucens* powder were obtained from Xuyang Insect Source Agricultural Development Co., Ltd. (Zhengzhou, China) and stored in a refrigerator at −20 °C for later use. A Bradford Protein Assay Kit was purchased from Solaibao Technology Co., Ltd. (Beijing, China). CE (S25111), PL (S10035), α-amylase (S31302), and α-glucosidase (S10050) were purchased from Yuanye Biotechnology Co., Ltd. (Shanghai, China). All other chemicals were of analytical grade and purchased from Sigma-Aldrich (Steinheim, Germany) and Macklin Biochemical Technology Co., Ltd. (Shanghai, China).

### 2.2. Experimental Insects

We hatched *A. domesticus* eggs after 7 days at 30 °C under controlled conditions. To standardize the larval population, we randomly selected 100 larvae for individual weight measurement (0.6 mg ± 0.02 mg) and subsequently allocated 5000 larvae equally into three replicate plastic containers (58 cm × 41 cm × 31 cm). Each container was provisioned with five vertically stacked cardboard egg trays to optimize spatial distribution. All experimental procedures were performed in an environmental chamber at 31 °C and 6% relative humidity throughout the trial period.

The experimental cricket feed was divided into 3 groups: pure plant feed, feed containing 5% BSFL, and feed containing 10% BSFL. The composition of the pure plant feed was prepared referring to [14] and is shown in Table 1. In the other two groups, soybean flour and semolina wheat flour were replaced by varying amounts of BSFL powder. The crude protein and fat levels and total energy of the feed were calculated according to the nutrition information of different components in the mixture, ensuring the nutrients of each feed group were basically equal. Experiments were conducted in duplicate for each group. Adequate feed and lettuce were provided during the feeding process, which was discontinued at 60 days, followed by killing with liquid nitrogen after starving for 48 h. Then, crickets were put in an oven at 60 °C for 48 h, ground for 30 s (YS–04 B, Beijing, China), weighed, and finally stored at −20 °C for subsequent use. The commercial house crickets and house crickets fed with pure plant feed are denoted by CHC and PPF, respectively, while 5% BSFL and 10% BSFL are used to represent the groups fed substrate containing 5% BSFL powder and 10% BSFL powder, respectively.

### 2.3. Proximate Composition

All nutritional parameters of *A. domesticus*, including protein, ash, and moisture, were measured according to the standard chemical composition analysis established by the Official Society of Analytical Chemists [15].

The total phenolic compounds (TPC) were extracted according to Zhang et al. [16] with slight modification. House cricket powder (0.5 g) was extracted twice with 70% ethanol (15 mL) under ultrasonication for 30 min. After centrifuging at 4 °C at 9000× *g* for 5 min, the pooled supernatant (1.0 mL) was mixed with 6.0 mL ddH_2_O and 1.0 mL of 50% Folin–Ciocalteu solution and incubated for 6 min at 25 °C. The mixture was then added to 4.0 mL of Na_2_CO_3_ solution (10.6 g/100 mL) and reacted at 30 °C for 60 min. The absorbance was measured at 760 nm using a Spectra Max Plus384 UV spectrophotometer (Molecular Devices, Sunnyvale, CA, USA). The results are expressed as mg gallic acid equivalent (mg GAE)/g.

The total chitin content was determined using the method of Huang et al. [17] with minor modifications. A filter bag with 1 g powder was stirred in NaOH solution (2M, 20 mL) for 5 h at 95 °C with continuous shaking (800 rpm). Then, the filter bag was placed in HCl solution (2M, 20 mL) and re-heated for 3 h at 50 °C. Finally, the chitin was dried in an air-dry oven for 36 h and retrieved.

### 2.4. Osborne Fractionation of Protein

Protein fractions of house cricket were determined based on the Osborne method [18]. Each sample (1 g) was sequentially extracted with 25 mL of ddH_2_O (pH = 6.0), 5% NaCl, 70% ethanol, and 0.1 M NaOH for albumin, globulin, prolamin, and glutenin, respectively. Solutions were stirred for 60 min at room temperature with continuous shaking (300 rpm). Each extract was centrifuged (centrifuge 5804, Eppendorf, Hamburg, Germany) at 5000× *g* for 10 min, and the supernatant was filtered through DMCS-treated glass wool. Each extraction step was repeated twice, and the supernatants were incorporated.

Albumin, globulin, and glutelin were obtained by adjusting the pH, while prolamin was obtained by adding a threefold volume of acetone. The precipitate proteins were allowed to rest for 6 h and centrifuged at 4000× *g* for 15 min. Subsequently, the precipitates were washed twice with ddH_2_O and neutralized pH before freeze–drying.

### 2.5. Total Amino Acid and Nutritional Quality Indices of Amino Acids

The amino acid profile was measured using an amino acid analyzer (L-8900, Hitachi, Tokyo, Japan) with a Na-cation-exchange column (200 mm × 4.6 mm). Each sample powder was added to 6M HCl, sealed, and hydrolyzed at 110 °C for 24 h. Then, the hydrolysate was diluted, dried, and deacidified. The residue was further dissolved in sample dilution buffer and filtered through a 0.22 μm filter. The total amino acid profile was subsequently determined using the external standard method of the amino acid analyzer.

The chemical score (CS) and essential amino acid index (EAAI) were calculated using the method recommended by Oser [19], and the essential amino acid score (AAS) was determined under the reference pattern by FAO/WHO/UNU [20]. The amino acid ratio coefficient (RC) and amino acid ratio coefficient score (SRC) were calculated according to the method of Li et al. [21]; the PDCAAS was determined for human nutrition only using the following formula, while the fecal true protein digestibility was estimated by referencing the result reported by Poelaert et al. [22]

PDCAAS (%)=fecal true protein digestibility× AAS of the first limited AA

### 2.6. Fatty Acid Composition

The fatty acid composition of *A. domesticus* was determined by gas chromatography. After methyl esterification, fatty acid methyl ester content was determined using a GC-7890B gas chromatograph (Agilent, Santa Clara, CA, USA) equipped with a flame-ionization detector (FID) and an SP-2330 capillary column (100 m × 0.25 mm × 0.20 μm film thickness). Referring to the parameters set by Andreadis et al. [23] during testing, the content of fatty acids was expressed as g/100 g total fatty acids identified.

The fatty acid compositions were further used to calculate lipid nutritional quality by atherogenicity indexes (AI) and thrombogenicity indexes (TI) [24].

$$\mathrm{AI}=\frac{C12:0+4\;\times \;C14:0+C16:0}{\sum \mathrm{MUFA}+\sum \text{n-6}+\sum \text{n-3}}$$

$$\mathrm{TI}=\frac{C14:0+C16:0+C18:0}{(0.5\;\times \;\sum \mathrm{MUFA})+(0.5\;\times \sum \text{n-6})+(3\;\times \;\sum \text{n-3})}$$

where ∑MUFA is the content of monounsaturated fatty acids; ∑n-3 and ∑n-6 are the content of omega-3 fatty acids and omega-6 fatty acids, respectively.

### 2.7. In Vitro Protein Digestion

In vitro gastrointestinal digestion was simulated based on the INFOGEST [25] protocol, with minor modifications. House cricket powder (1 g) was added to 10 mL of simulated salivary fluid (without amylase) to simulate oral digestion for 2 min. Then, gastric digestion was simulated by adding 10 mL of simulated gastric fluid (including 20,000 U of pepsin), decreasing the pH to 3.0 with 1 M HCl. After incubation at 37 °C for 2 h using a lab shaker (TS-200B, JTLY, Changzhou, China), intestinal digestion was initiated by adding 10 mL of simulated intestinal solution (including 2000 U of pancreatin and 0.32 g of fresh bile) and the pH was raised to 7.0 with 1 M NaOH. The mixture was also incubated at 37 °C for 2 h with continuous shaking. Digestion was terminated by placing the mixture in boiling water for 10 min.

During the process of simulation digestion, digests (2 mL) were collected at 0, 30, 60, 90, and 120 min of the intestinal phase. The digests were boiled for 10 min to stop the reaction and centrifuged at 5000× *g* for 15 min. The supernatants were collected, kept in plastic tubes, and stored at −20 °C for further analysis. Protein digestibility was determined according to the method of Li et al. [26], through which the ratio of protein content in the digests to the protein content of *A. domesticus* is measured using a Bradford assay.

### 2.8. Degree of Hydrolysis (DH%)

We added 0.83 mL of the TCA solution (5%) to 0.5 mL of the digested sample and centrifuged at 10,000× *g* for 30 min to precipitate the undigested protein [27]. The degree of hydrolysis was quantified by monitoring the proportion of free amino group concentration in the digests to total amino group concentration in house cricket. The free amino group concentration in the digests was measured via an *o*-phthaldialdehyde (OPA) spectrophotometric assay according to the method of Zahir et al. [28] with some modifications. The fresh OPA solution was prepared before each experiment as follows: we dissolved 7.62 g of disodium tetraborate decahydrate in 150 mL of ddH_2_O. Then, 4 mL of ethanol solution, which contains 160 mg of *o*-phthaldialdehyde, 200 mg of sodium dodecyl sulfate, 176 mg of dithiothreitol, and 46 mL of ddH_2_O, were added to the above solution and further mixed. The OPA solution (3 mL) was combined with the digested sample (0.4 mL) at room temperature for 2 min, followed by measurement at 340 nm. The absorbance was further compared with the standard curve of L-serine (12.5~100 mg/L) to determine the concentration of free amino groups in each sample. The total amino group concentration in house crickets was determined using the hydrolysate of HCl prepared in Section 2.4.

### 2.9. Antioxidant Activity In Vitro

#### 2.9.1. DPPH Radical Scavenging Activity

The DPPH radical scavenging activity of the digests of *A. domesticus* protein was determined according to the method of Zahir et al. [28] with a minor modification. Different concentrations of protein hydrolysate (1.0 mL, 0.2~2.0 mg/mL) were incubated with 2.0 mL DPPH–ethyl alcohol solution (0.2 mM) in the dark at 37 for 30 °C min. The absorbance was measured at 517 nm and ddH_2_O was utilized as a substitute for the sample in the black group. The results were calculated as follows:

$$\mathrm{DPPH}\;\mathrm{radical}\;\mathrm{scavenging}\;\mathrm{activity}\;(\%)=\left[1-\frac{A_{1}-A_{2}}{A_{0}}\right]\times 100$$

where A_1_ is the absorbance of the sample, A_2_ is the absorbance of the sample with ethyl alcohol solution (without DPPH), and A_0_ is the absorbance of the blank.

#### 2.9.2. ABTS Radical Scavenging Activity

The ABTS radical scavenging activity was determined by the method reported by Zahir et al. [28] with minor modifications. A total of 5 mL of 7 mM ABTS and 0.88 μL of 2.5 mM K_2_S_2_O_8_ were mixed to form the ABTS working solution and incubated in the dark for 14 h. Moreover, 1.0 mL of protein hydrolysate at different concentrations (0.2~2.0 mg/mL) was incubated with 2.0 mL of ABTS solution in the dark at 37 °C for 30 min. The absorbance was measured at 734 nm, and the result of ABTS radical scavenging activity was similar to the results expressed by DPPH.

#### 2.9.3. Hydroxide Radical (OH^−^) Scavenging Activity

The OH^−^ scavenging activity was determined according to the method of Zhang et al. [29]. Different concentrations of protein hydrolysate (2 mL, 0.2~2.0 mg/mL) were mixed with 0.5 mL of 6 mM FeSO_4_ solution, 0.5 mL of 6 mM salicylic acid–ethanol solution, and 1.5 mL ddH_2_O. Then, we added 0.5 mL of 6 mM H_2_O_2_ solution, shook it evenly, and incubated it at 37 °C for 30 min. The absorbance was measured at 536 nm, and the results were calculated as follows:

$${\mathrm{OH}}^{-}\;\mathrm{scavenging}\;\mathrm{activity}\;(\%)=\left[\frac{A_{1}-A_{0}}{A_{2}-A_{0}}\right]\times \;100$$

where A_1_ is the absorbance of the sample, A_2_ is the absorbance of the sample with ddH_2_O (without H_2_O_2_), and A_0_ is the absorbance of the blank.

#### 2.9.4. Potassium Hexacyanoferrate (III) Total Reducing Power Assay (TRP)

The TRP assay was conducted using the method of Zhang et al. [29]. The TRP reaction system was prepared by adding 1.0 mL of K_3_[Fe(CN)_6_] solution (10%) and 2.5 mL of PBS (0.2 M, pH 6.6) and then incubated at 50 °C for 20 min. We mixed different concentrations of protein hydrolysate (0.1 mL, 0.2~2.0 mg/mL) and 2 mL of the reaction solution. After cooling at room temperature, we added 2.5 mL TCA solution (10%) and centrifuged it at 3000 × g for 10 min to obtain the supernatant. Then, 2.5 mL of supernatant, 0.45 mL of FeCl_3_ solution, and 2 mL of ddH_2_O were mixed and incubated for 2 min. Distilled water was used instead of the sample in the blank control group. The absorbance was measured at 700 nm, and the result was calculated as blank absorbance minus sample absorbance.

#### 2.9.5. Ferric Reducing Antioxidant Power (FRAP)

The FRAP assay was performed according to the method reported by Ribeiro et al. [30] with minor modifications. The FRAP working solution was prepared by combining 0.3 M acetate buffer, 10 mM TPTZ, and 20 mM FeCl_3_ in a volumetric ratio of 10:1:1. Then, 290 μL of the working solution was added to the different concentrations of protein hydrolysate (0.5 mL, 0.2~2.0 mg/mL) and incubated for 5 min at 37 °C in the darkness. The absorbance was measured at 593 nm, and the result of FRAP was similar to the results expressed by TRP.

#### 2.9.6. Cupric Ion-Reducing Activity (CUPRAC)

The CUPRAC assay was measured as follows [29]: different concentrations of protein hydrolysate (0.5 mL, 0.4~4.0 mg/mL) were added to 3.0 mL of reaction mixture containing 1.0 mL of CuCl_2_ (10.0 mM), 1.0 mL of neocupric (7.5 mM) and 1.0 mL of CH_3_COONH_4_ solution (1.0 M). The reaction without CuCl_2_ was used to prepare the blank solution. The absorbance was measured at 450 nm, and the FRAP result was similar to the results expressed by TRP.

### 2.10. Statistical Analysis

All tests were performed in triplicate. SPSS statistical software (20.0.0, IBM, Chicago, IL, USA) was used to perform analysis of variance (ANOVA) on data, followed by Duncan’s test to assess the significant difference between means (*p* < 0.05). Experimental data were visualized in OriginPro 2021 software (9.8.0.200, OLC, Northampton, MA, USA) and GraphPad Prism (8.0.1, GPS, San Diego, CA, USA).

## 3. Results and Discussion

### 3.1. Yield and Proximate Analysis

After 60 days of feeding, most *A. domesticus* in all groups were adults. Compared with the group fed on pure plant feed, more cricket powder was harvested in the 5% BSFL and 10% BSFL groups; in particular, the mass produced by the 5% BSFL group increased by 23.79% (Table 2). This was primarily attributed to variations in feed composition and the survival rate of crickets. Previous studies have shown that adding BSFL can change the palatability of the feed and effectively enhance the disease resistance of animals [31]. Furthermore, the yield of house crickets can also be influenced by various factors such as developmental stages, sex ratio, and body weight.

The commercial house cricket (CHC) powder exhibited higher levels of moisture, crude fat, ash, and chitin in its nutrient composition, potentially because of the distinct large-scale feeding methods. Compared with PPF, the 5% BSFL and 10% BSFL groups exhibited higher crude protein and total phenolic compound (TPC) contents but lower moisture and crude fat contents. The ash and chitin contents were not significantly different (*p* = 0.167). The higher protein content can be attributed to the increased digestibility and absorptivity of animal protein [32], with house crickets more easily converting BSFL protein into their own protein. The reduction in fat may be related to the developmental stage and gender ratio, as nymphs and females tend to have higher fat contents [33,34,35]. BSFL also has the effect of lowering blood lipids, which will also lead to reduced fat content in crickets [36]. The content of TPC also showed a positive correlation with the amount of BSFL. Additionally, these measured nutrient values fell within the range of previous measurements [37], suggesting that the dietary intervention had minimal impact on the nutrient composition of crickets and indicating a relatively stable nutrient content in adult crickets.

### 3.2. Osborne Fractionation

**Figure 1:**
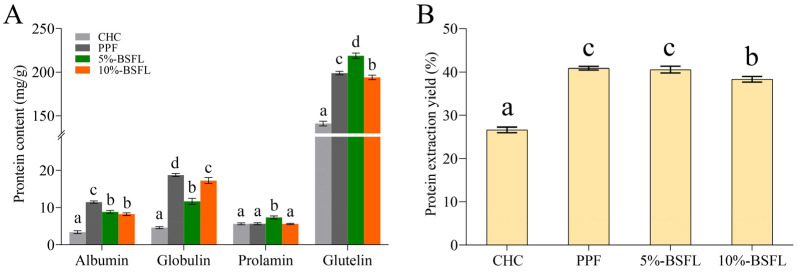
Concentration of protein fractions in *A. domesticus* reared on different feeds. Protein content (**A**), Protein extraction yield (**B**). CHC: commercial house cricket; PPF: house cricket fed with pure plant feed; 5% BSFL: the group fed substrate containing 5% BSFL powder; 10% BSFL: the group fed substrate containing 10% BSFL powder. Different letters within each column indicate significant differences (*p* < 0.05) between mean values, as determined by Duncan’s test.

The results of Osborn fractionation and the extraction rate of total protein are shown in Figure 1 and Tables S1 and S2. The crickets reared in the laboratory exhibited a significant improvement in extraction rate compared with CHC (*p* < 0.001), though their crude protein content was similar. This may be due to the different isoelectric points of commercial house cricket protein. Among the other three crickets, PPF exhibited the highest levels of albumin and globulin, while the 5% BSFL group showed the highest content of prolamin and glutelin. These results indicate that different feeds can alter the protein composition of crickets, with the addition of BSFL powder leading to increased levels of alcohol- and alkali-soluble proteins in house crickets. However, it is important to note that these changes are relatively restricted in scope, suggesting that this method has only a limited impact on the protein composition.

Our results were different from those of Stone et al. [38], and this may be related to the species, methods of protein measurement, and life cycles of crickets. The analysis revealed that the amount of total protein in 5% BSFL (40.53 ± 0.76%) and 10% BSFL (38.32 ± 0.66%) groups was lower than that of PPF (40.87 ± 0.41%), indicating that a change in feed could impact the efficiency of house cricket protein extraction, but the increased yield might compensate for this limitation. Furthermore, the house crickets’ protein composition predominantly consisted of glutelin (84.76 ± 0.02~91.21 ± 0.38%) (Table S2), which explains why researchers prefer to use dilute alkaline solutions to extract proteins from crickets.

### 3.3. Free Amino Acid (FAA) Profile

The free amino acid compositions of CHC and different *A. domesticus* reared on various feed types are presented in Table 3. Glutamic (106.89 ± 2.00~115.39 ± 2.07 mg/g), alanine (91.31 ± 1.30~114.93 ± 1.55 mg/g), and aspartic (71.99 ± 1.31~81.58 ± 4.35 mg/g) acids emerged as the predominant free amino acids in house crickets, while cysteine (5.24 ± 0.00~6.31 ± 0.78 mg/g), methionine (12.61 ± 1.51~14.40 ± 0.00 mg/g), and histidine (25.94 ± 1.69~27.04 ± 1.17 mg/g) exhibited lower abundances. Furthermore, the proportions of various amino acids were similar to those in previous studies [39], indicating that the composition of amino acids in *A. domesticus* was relatively stable. The impact of different feed and growth environments on the amino acid composition was not significant. After adding BSFL to the feed, the content of four essential amino acids—threonine, valine, isoleucine, and phenylalanine—in the 5% BSFL and 10% BSFL groups increased by 1.06~5.59%, 7.38~7.80%, 2.64~8.66%, and 2.13~8.67%, respectively (*p* < 0.001), which led to a direct increase in the content of essential amino acids (EAAs). Similarly, the AAS, CS, and RC of most essential amino acids also improved accordingly (Table S3). This was primarily due to the higher concentration of essential amino acids in animal-source feed compared with plant-source feed [40]. The essential amino acids in BSFL, which are present in high amounts, can convert into endogenous amino acids in the crickets, thereby enhancing house crickets’ nutritional quality.

According to the different evaluation methods, the first limiting amino acid was Met + Cys or Met (Table S3), which was also found in previous studies [39]. However, several studies have also demonstrated that the first limiting amino acid of *A. domesticus* is leucine or lysine [22]. These results indicate that different calculation methods, species, and feeding methods affect the limiting amino acid and amino acid composition, and the difference is mainly reflected in essential amino acids or low-content amino acids. Furthermore, the addition of BSFL did not yield a significant increase in the Met content within the house crickets, which might be related to their amino acid composition. This value could be increased by adding other substances in subsequent experiments. In another evaluation method of amino acids, the EAAI value of 5% BSFL and 10% BSFL groups showed a significant increase (*p* = 0.035), while the changes in SRC (*p* = 0.667) and PDCAAS (*p* = 0.735) were not statistically significant (Table 3). This suggests that there was a certain degree of alteration in amino acid content, but it did not effectively enhance the nutritional value of amino acids.

### 3.4. Fatty Acids Profile

The fatty acid compositions of different *A. domesticus* reared on different feeds are summarized in Table 4. Statistical differences were found in most fatty acids between different samples, although most of the measurements were within the range of previous measurements [37]. The predominant fatty acids in house cricket were found to be palmitic acid, oleic acid, and linoleic acid, collectively accounting for over 80% of the total content. In CHC, the samples exhibited a significantly higher content of palmitic acid (29.39 ± 0.02%) and oleic acid (24.42 ± 0.03%) compared with the house crickets reared in our study (palmitic acid: 14.64 ± 0.09~14.98 ± 0.22%; oleic acid: 17.78 ± 0.33~19.97 ± 0.16%). However, the concentration of linoleic acid (29.76 ± 0.03%) exhibited a significant decrease (*p* < 0.001) (PPF: 50.82 ± 0.25%; 5% BSFL: 51.38 ± 0.11%; 10% BSFL: 50.97 ± 0.56%). This is related to the composition of the feed. The main components in our feed were soybean, corn, and whole wheat flour, all of which contain abundant linoleic acid [41,42,43]. In addition, the reduction in linoleic acid content led to an elevation in the proportion of other fatty acids, particularly palmitic acid, stearic acid, and linoleic acid, which may be attributed to the increased inclusion of soybean meal and wheat bran in the commercial cricket feed [44]. Differences in the types of fatty acids in commercial crickets were also detected (lauric acid, pentadecanoic acid, arachidic acid, and eicosapentaenoic acid were detected, while dohom-gamma-linolenic acid was not detected), which may be related to specific ingredients or different treatments in the feed.

The fatty acid composition in the crickets we reared exhibited minimal changes. The inclusion of BSFL resulted in the detection of a small amount of linoleic trans acid in the 5% BSFL (0.17 ± 0.01%) and 10% BSFL (0.20 ± 0.02%) groups while significantly enhancing the levels of myristic acid (5% BSFL: 0.39 ± 0.00%; 10% BSFL: 0.57 ± 0.01%), palmitic acid (5% BSFL: 14.98 ± 0.22%; 10% BSFL: 14.92 ± 0.09%), palmitoleic acid (5% BSFL: 0.58 ± 0.04%; 10% BSFL: 0.86 ± 0.01%), and oleic acid (5% BSFL: 17.88 ± 0.25%; 10% BSFL: 19.97 ± 0.16%) compared with the PPF (myristic acid: 0.33 ± 0.01%; palmitic acid: 14.64 ± 0.09%; palmitoleic acid: 0.47 ± 0.02%; oleic acid: 17.78 ± 0.33%) (*p* < 0.001). This suggests that house crickets were more likely to absorb fatty acids from BSFL than plants.

After incorporating BSFL into the feed, there was an increase in the levels of saturated fatty acids (SFAs) and monounsaturated fatty acids (MUFAs) in the house crickets, while the concentrations of polyunsaturated fatty acids (PUFAs), n-3, and n-3/n-6 declined. This phenomenon was also observed in experiments involving BSFL as aquatic feed, which led to an increase in the total n-3 PUFA content and a reduction in the total n-6 PUFA content in the muscles of aquatic organisms [45,46]. Although BSFL is known to be rich in lauric acid, the analysis of the 5% BSFL and 10% BSFL groups revealed a minimal content of lauric acid (10% BSFL: 0.19 ± 0.00%). This suggests that house crickets may not effectively accumulate lauric acid and instead readily utilize it for energy production through oxidation [47].

Finally, the AI and TI values were employed to assess the nutritional quality of fatty acids. Our results demonstrate that lab-raised crickets exhibited superior suitability for human consumption; the addition of BSFL did not yield a significant reduction in these indices.

### 3.5. Protein Digestibility and DH (%) of Protein

**Figure 2:**
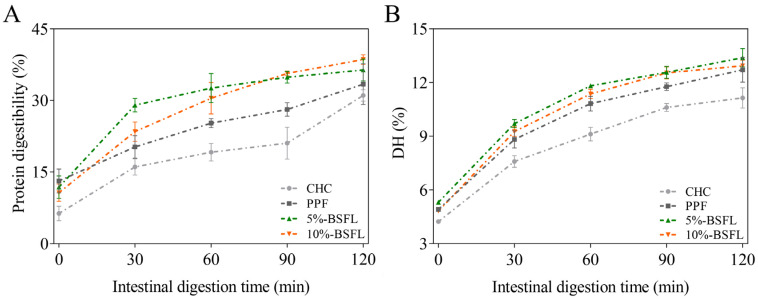
Protein digestibility (**A**) and hydrolysis degree (**B**) of protein in *A. domesticus* reared on different feeds during in vitro digestion. CHC: commercial house cricket; PPF: house cricket fed with pure plant feed; 5% BSFL: the group fed substrate containing 5% BSFL powder; 10% BSFL: the group fed substrate containing 10% BSFL powder.

The aforementioned findings confirmed the variations in nutrient composition among different house crickets, particularly with regard to crude protein content, amino acid composition, and protein components. These disparities may consequently contribute to varying levels of protein digestibility across various cricket types. As shown in Figure 2 and Table S4, after gastric digestion, the digestibility of PPF (13.13 ± 2.50%) was significantly higher than that of the other three groups (*p* = 0.03). The digestibility of 5% BSFL (11.82 ± 2.38%) and 10% BSFL (10.66 ± 1.82%) exhibited similar values, while CHC (6.31 ± 1.52%) demonstrated the lowest digestibility. This discrepancy may be attributed to variances in protein solubility rather than differences in gastric protease hydrolysis [48]. In addition, pepsin could enzymatically hydrolyze proteins into peptide fragments; however, the small intestine serves as the primary site for protein digestion, where pancreatin, a complex enzyme, further hydrolyzes peptide chains into smaller peptides or amino acids [49]. Therefore, our main focus was on monitoring changes that occur during the intestinal digestion stage.

During intestinal digestion, the highest rate of protein digestion was exhibited in the initial 30 min period, with significantly higher protein digestibility observed for the 5% BSFL (29.01 ± 1.41%) and 10% BSFL (23.46 ± 2.03%) groups compared with the CHC (16.07 ± 1.69%) and PPF (20.27 ± 2.37%) groups. Following intestinal digestion, the protein digestibility was found to be as follows: CHC: 31.06 ± 1.21%, PPF: 33.41 ± 4.26%, 5% BSFL: 36.37 ± 2.29%, and 10% BSFL: 38.59 ± 1.00%. This suggests that the inclusion of BSFL in the diet may influence the digestibility of cricket protein, potentially attributed to variations in molecular structures among proteins. The protein in CHC might possess higher molecular weights and require longer digestion times, which is also indicated by the increased digestion rate of 90–120 min. The PDCAAS results indicate that the nutritional quality of crickets given feed supplemented with 5% BSFL and 10% BSFL was marginally higher than that of the CHC and PPF groups, but the difference was not statistically significant [26].

The hydrolysis of peptide bonds in cricket protein was assessed using DH (%). The hydrolysis degree of commercial house crickets (CHC) was the lowest (4.22 ± 0.07~11.14 ± 0.57%), followed by PPF (4.91 ± 0.06%~12.71 ± 0.69%). The proteins in the 5% BSFL (5.31 ± 0.03%~13.38 ± 0.51%) and 10% BSFL (4.82 ± 0.03%~12.93 ± 0.31%) groups demonstrated consistently high rates of hydrolysis throughout the digestive stage, even at the termination of digestion. This suggests that house crickets fed on BSFL may exhibit enhanced peptide and amino acid production during digestion, rendering them more suitable for generating protein hydrolysates. Our values are lower than those reported by Hall et al. [13], which may be attributed to the different enzyme types, enzyme concentrations, reaction conditions, and detection methodologies. However, our findings demonstrate that house cricket protein has a higher DH than wheat bran protein [26], possibly due to the fact that animal proteins are better hydrolyzed than plant proteins. The hydrolysis of amino acids is a time-consuming process, typically necessitating the use of protease or strong acid solutions and high temperatures to achieve completion [50]. When utilizing protease, the enzyme concentration and the reaction time are pivotal factors influencing hydrolysis. There was little difference in DH among the groups. Previous research has indicated that the hydrolysis degree is primarily associated with the particle size of the substrate and protein type. Generally, smaller substrate particle sizes and purified proteins lead to higher degrees of hydrolysis [26].

### 3.6. Antioxidant Properties

**Figure 3:**
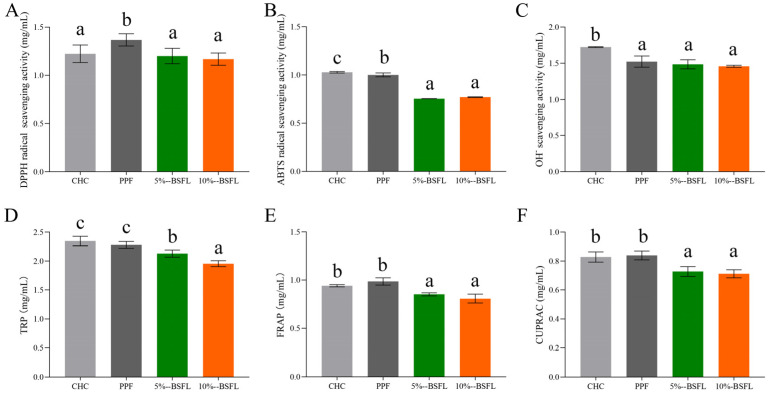
The antioxidant activity of proteins in different *A. domesticus* during in vitro digestion. DPPH radical scavenging activity (**A**), ABTS radical scavenging activity (**B**), hydroxide radical (OH-) scavenging activity (**C**), potassium hexacyanoferrate (III) total reducing power assay (TRP) (**D**), ferric reducing antioxidant power (FRAP) (**E**), cupric ion reducing activity (CUPRAC) (**F**). CHC: commercial house cricket; PPF: house cricket fed with pure plant feed; 5% BSFL: the group fed substrate containing 5% BSFL powder; 10% BSFL: the group fed substrate containing 10% BSFL powder. Different letters within each column indicate significant differences (*p* < 0.05) between mean values, as determined by Duncan’s test.

After digestion, cricket protein can generate a large number of peptides and free amino acids [51]. Apart from their nutritional value, these small bioactive molecules can also produce hormone-like effects on physiological processes, thereby influencing metabolic or physiological pathways and contributing to human health [52]. Based on variations in the number and types of peptides and amino acids present in cricket protein hydrolysate, a single index is inadequate for accurately reflecting their antioxidant effect. Therefore, we determined six antioxidant indexes during cricket protein digestion through in vitro experiments. The results are shown in Figure 3 and Table S5.

The IC50 values of CHC exhibited the highest value in ABTS radical scavenging activity, OH^-^ scavenging activity, and TRP, whereas PPF demonstrated the highest IC50 values for DPPH radical scavenging activity, TRAP and CUPRAC. In the three groups of crickets we reared, the antioxidant activity of protein hydrolysate in the 5% BSFL and 10% BSFL groups was found to be stronger than that of PPF. This indicates that the protein hydrolysate in commercial house crickets and those fed with pure plant feed exhibited a lower antioxidant activity compared with that of crickets raised on feed supplemented with BSFL powder. This may be due to variations in the composition and structure of cricket proteins, leading to discrepancies in the composition of the hydrolysate. The antioxidant activity of hydrolysate is mainly related to the molecular weight, amino acid composition, sequence, and secondary structure of hydrolyzed peptides [53]. Polypeptides with a lower molecular weight (<3 kDa) and an increased proportion of hydrophobic amino acid residues tend to exhibit enhanced antioxidant activity [54]. In addition, antioxidant activity is also influenced by the composition of amino acids [55]. The 5% BSFL and 10% BSFL groups contained more antioxidant amino acids, such as Tyrosine and phenylalanine, which validated the high antioxidant activity.

Previous studies have demonstrated that substituting fishmeal with BSFL can enhance the antioxidant capacity of basa and grass carp [56,57], which indicates that incorporating BSFL into feed formulations could effectively improve the antioxidant activity of organisms. Moreover, proteolysis may further enhance antioxidant activity. However, with the exception of TRP, no statistically significant differences were observed between the 5% BSFL and 10% BSFL groups, indicating that the level of addition had little impact on the antioxidant activity. As a natural and safe source of antioxidants in food, digestion-derived antioxidant peptides can effectively reduce reactive oxygen species (ROS) production, enhance free radical scavenging activity, and fortify endogenous enzyme defense systems as well as non-enzymatic antioxidants [58]. Our results show that the proteolytic substance of house crickets fed with BSFL had a higher antioxidant activity, which can lead to the production of products more suitable for human consumption.

## 4. Conclusions

This study explored the impact of supplementing feed with BSFL on the yield, nutritional composition, and protein digestion characteristics of *Acheta domesticus*. In addition, the bioactivity of protein hydrolysate in house crickets was compared. The results suggest that feeding house crickets with a small amount of BSFL could enhance their yield and crude protein and total phenolic contents while also altering their amino acid and fatty acid profiles. During the process of digestion, the protein digestibility of 5% BSFL and 10% BSFL was higher. In terms of the in vitro bioactivity activity, the protein hydrolysate from 5% BSFL and 10% BSFL groups demonstrated a higher antioxidant activity. In conclusion, the addition of BSFL to feed effectively enhanced the yield and nutritional characteristics of house crickets. After consumption, the protein became more readily digestible. Moreover, their protein hydrolysate exhibited a greater potential to prevent and treat body damage caused by oxidative stress and metabolic disorders.

Incorporating animal-derived ingredients into feed can positively influence the growth of crickets, thereby enhancing their potential as a sustainable food source. Subsequently, we will further explore the utilization of high-quality crickets for protein concentrate and functional peptide preparation, as well as evaluate their physicochemical properties and functional characteristics both in vivo and in vitro.

## Figures and Tables

**Table 1:** Composition of experimental feed.

	Corn (g/100 g)	Soybean (g/100 g)	Whole Wheat (g/100 g)	Yeast (g/100 g)	*H. illucens* (g/100 g)	Protein (g/100 g)	Fat (g/100)	Energy (J/100 g)
PPF	25	45	25	5	0	23.4	10.10	1449.20
5% BSFL	30	38	22	5	5	23.36	10.11	1457.28
10% BSFL	34	31	20	5	10	23.41	10.12	1462.52

PPF, crickets fed on pure plant feed; 5% BSFL, the group fed substrate containing 5% of BSFL powder added feed; 10% BSFL, the group fed substrate containing 10% of BSFL powder.

**Table 2:** The yield and proximate analysis of *A. domesticus* reared on different feeds.

	CHC	PPF	5% BSFL	10% BSFL	*p*-Value
Yield (g)	—	129.37 ± 12.12 ^a^	169.76 ± 3.42 ^c^	146.81 ± 6.42 ^b^	<0.001
Moisture (%)	10.68 ± 0.69 ^d^	6.39 ± 0.31 ^c^	5.49 ± 0.11 ^a^	5.59 ± 0.10 ^b^	<0.001
Fat (%)	24.19 ± 0.17 ^c^	22.90 ± 0.23 ^b^	20.38 ± 0.13 ^a^	20.56 ± 0.74 ^a^	<0.001
Protein (%)	58.12 ± 0.32 ^ab^	57.47 ± 0.53 ^a^	60.83 ± 0.74 ^c^	58.75 ± 0.11 ^b^	<0.001
Ash (%)	4.83 ± 0.01 ^b^	4.50 ± 0.01 ^a^	4.52 ± 0.04 ^a^	4.50 ± 0.03 ^a^	<0.001
Chitin (%)	8.32 ± 0.33	7.62 ± 0.11	7.80 ± 0.60	7.86 ± 0.09	0.167
TPC (mg/g)	11.93 ± 0.17 ^ab^	11.43 ± 0.05 ^a^	12.10 ± 0.44 ^b^	12.93 ± 0.48 ^c^	0.004

The experiment was carried out in triplicate (*n* = 3). Superscripts of different letters within each column indicate significant differences (*p* < 0.05) between mean values, as determined by Duncan’s test. CHC: commercial house cricket; PPF: house cricket fed with pure plant feed; 5% BSFL: the group fed substrate containing 5% BSFL powder; 10% BSFL: the group fed substrate containing 10% BSFL powder; TPC: total phenolic compound.

**Table 3:** Total amino acid profile (mg/g protein), EAAI, SRC, and PDCAAS of *A. domesticus* reared on different feeds.

	CHC	PPF	5% BSFL	10% BSFL	*p*-Value
Threonine	36.96 ± 0.75 ^a^	37.56 ± 1.34 ^a^	37.96 ± 0.00 ^a^	39.66 ± 0.78 ^b^	0.025
Valine	45.65 ± 0.00 ^a^	47.85 ± 2.79 ^ab^	51.48 ± 0.76 ^b^	51.38 ± 1.35 ^b^	0.05
Methionine	12.61 ± 1.51	12.97 ± 0.78	14.40 ± 0.00	13.07 ± 3.40	0.687
Isoleucine	31.74 ± 0.75 ^a^	34.43 ± 1.55 ^b^	35.34 ± 1.31 ^bc^	37.41 ± 0.78 ^c^	0.002
Leucine	67.39 ± 0.75 ^a^	70.21 ± 3.38 ^ab^	66.75 ± 1.31 ^a^	73.02 ± 1.35 ^b^	0.016
Phenylalanine	26.96 ± 0.75 ^a^	28.62 ± 2.05 ^ab^	29.23 ± 0.76 ^ab^	31.10 ± 1.35 ^b^	0.032
Histidine	26.09 ± 0.00	25.94 ± 1.69	25.96 ± 0.38	27.04 ± 1.17	0.543
Lysine	47.39 ± 0.75	47.40 ± 3.10	48.43 ± 1.31	49.13 ± 0.78	0.583
EAA	294.79 ± 1.30 ^a^	304.98 ± 14.79 ^ab^	309.55 ± 5.59 ^ab^	321.82 ± 3.38 ^b^	0.022
Arginine	67.83 ± 4.52 ^b^	64.84 ± 0.78 ^b^	52.79 ± 1.51 ^a^	55.44 ± 4.88 ^a^	0.002
Proline	56.52 ± 0.75 ^a^	58.13 ± 2.05 ^ab^	62.83 ± 3.46 ^b^	62.65 ± 2.82 ^b^	0.031
Aspartic	80.00 ± 1.51 ^b^	80.94 ± 4.31 ^b^	71.99 ± 1.31 ^a^	81.58 ± 4.35 ^b^	0.021
Serine	48.26 ± 0.00 ^b^	52.77 ± 2.05 ^c^	41.45 ± 0.76 ^a^	54.09 ± 1.35 ^c^	<0.001
Glutamic	108.70 ± 2.72	110.9 ± 6.20	106.89 ± 2.00	115.39 ± 2.07	0.096
Glycine	56.52 ± 1.51 ^a^	65.29 ± 0.78 ^b^	64.14 ± 0.00 ^b^	63.10 ± 1.56 ^b^	<0.001
Alanine	91.31 ± 1.30 ^a^	114.93 ± 1.55 ^b^	113.00 ± 3.78 ^b^	110.43 ± 2.82 ^b^	<0.001
Cystine	5.65 ± 0.75	5.37 ± 1.34	5.24 ± 0.00	6.31 ± 0.78	0.472
Tyrosine	44.78 ± 0.75 ^a^	45.17 ± 2.79 ^a^	56.72 ± 0.76 ^b^	47.33 ± 1.35 ^a^	<0.001
NEAA	491.75 ± 4.70 ^a^	533.5 ± 14.47 ^b^	522.25 ± 4.53 ^b^	540.87 ± 15.36 ^b^	0.003
TAA	854.36 ± 1.3 ^a^	903.32 ± 28.95 ^b^	884.59 ± 9.49 ^ab^	918.12 ± 22.80 ^b^	0.017
EAAI	56.82 ± 0.76 ^a^	58.65 ± 2.63 ^ab^	59.96 ± 0.99 ^ab^	61.38 ± 1.04 ^b^	0.035
SRC	0.27 ± 0.01	0.26 ± 0.03	0.27 ± 0.00	0.25 ± 0.03	0.667
PDCAAS	56.74 ± 4.05	56.97 ± 6.37	61.01 ± 0.00	60.22 ± 8.75	0.735

The experiment was carried out in triplicate (*n* = 3). Superscripts of different letters within each column indicate significant differences (*p* < 0.05) between mean values, as determined by Duncan’s test. CHC: commercial house cricket; PPF: house cricket fed with pure plant feed; 5% BSFL: the group fed substrate containing 5% BSFL powder; 10% BSFL: the group fed substrate containing 10% BSFL powder; EAAs: essential amino acids; NEAAs, nonessential amino acids; TAAs: total amino acids; EAAI: essential amino acid index; SRC: amino acid ratio coefficient score; PDCAAS: protein digestibility-corrected amino acid score.

**Table 4:** Total Fatty acid composition (%), AI, and TI of *A. domesticus* reared on different feeds.

	CHC	PPF	5% BSFL	10% BSFL	*p*-Value
Lauric acid (C12:0)	0.34 ± 0.00 ^b^	ND	ND	0.19 ± 0.00 ^a^	<0.001
Myristic acid (C14:0)	0.65 ± 0.01 ^d^	0.33 ± 0.01 ^a^	0.39 ± 0.00 ^b^	0.57 ± 0.01 ^c^	<0.001
Pentadecanoic acid (C15:0)	0.13 ± 0.01	ND	ND	ND	-
Palmitic acid (C16:0)	29.39 ± 0.02 ^c^	14.64 ± 0.09 ^a^	14.98 ± 0.22 ^b^	14.92 ± 0.09 ^b^	<0.001
Palmitoleic acid (C16:1n7)	0.63 ± 0.01 ^c^	0.47 ± 0.02 ^a^	0.58 ± 0.04 ^b^	0.86 ± 0.01 ^d^	<0.001
Heptadecanoic acid (C17:0)	0.21 ± 0.00 ^b^	0.19 ± 0.02 ^ab^	0.17 ± 0.02 ^ab^	0.16 ± 0.02 ^a^	0.042
Stearic acid (C18:0)	12.11 ± 0.01 ^c^	7.52 ± 0.14 ^b^	7.49 ± 0.11 ^ab^	7.28 ± 0.14 ^a^	<0.001
Oleic trans acid (C18:1n9t)	0.13 ± 0.02 ^a^	1.48 ± 0.09 ^d^	0.88 ± 0.06 ^c^	0.75 ± 0.04 ^b^	<0.001
Oleic acid (C18:1n9c)	24.42 ± 0.03 ^c^	17.78 ± 0.33 ^a^	17.88 ± 0.25 ^a^	19.97 ± 0.16 ^b^	<0.001
Linoleic trans acid (C18:2n6t)	ND	ND	0.20 ± 0.02 ^b^	0.17 ± 0.01 ^a^	0.063
Linoleic acid (C18:2n6c)	29.76 ± 0.03 ^a^	50.82 ± 0.25 ^b^	51.38 ± 0.11 ^b^	50.97 ± 0.56 ^b^	<0.001
γ-Linolenic acid (C18:3n6)	0.24 ± 0.01	0.25 ± 0.03	0.20 ± 0.01	ND	0.442
α-Linolenic acid (C18:3n3)	1.10 ± 0.01 ^a^	4.27 ± 0.06 ^c^	3.4 ± 0.11 ^b^	3.38 ± 0.03 ^b^	<0.001
Arachidic acid (C20:1)	0.18 ± 0.01 ^a^	ND	ND	1.18 ± 0.05 ^b^	<0.001
Dohomo–γ–Linolenic Acid(C20:3n6)	ND	1.10 ± 0.03 ^b^	1.25 ± 0.04 ^b^	0.81 ± 0.12 ^a^	0.001
Eicosapentaenoic Acid (C20:5n3)	0.73 ± 0.04 ^a^	1.14 ± 0.09 ^b^	ND	ND	0.002
SFA	42.82 ± 0.02 ^c^	22.68 ± 0.11 ^a^	23.04 ± 0.28 ^b^	22.93 ± 0.13 ^b^	<0.001
MUFA	25.36 ± 0.02 ^d^	19.73 ± 0.27 ^a^	20.52 ± 0.19 ^b^	21.58 ± 0.18 ^c^	<0.001
PUFA	31.82 ± 0.01 ^a^	57.58 ± 0.21 ^d^	56.44 ± 0.16 ^c^	55.53 ± 0.42 ^b^	<0.001
n-3	1.10 ± 0.01 ^a^	4.27 ± 0.06 ^c^	3.40 ± 0.11 ^b^	3.38 ± 0.03 ^b^	<0.001
n-6	30.00 ± 0.04 ^a^	52.17 ± 0.24 ^b^	53.04 ± 0.13 ^c^	52.16 ± 0.39 ^b^	<0.001
n-3/n-6	0.04 ± 0.00 ^a^	0.08 ± 0.00 ^c^	0.06 ± 0.00 ^b^	0.06 ± 0.00 ^b^	<0.001
AI	0.54 ± 0.00 ^c^	0.20 ± 0.00 ^a^	0.20 ± 0.00 ^ab^	0.20 ± 0.00 ^b^	<0.001
TI	1.35 ± 0.00 ^c^	0.45 ± 0.00 ^a^	0.48 ± 0.01 ^b^	0.47 ± 0.00 ^b^	<0.001

The experiment was carried out in triplicate (*n* = 3). Superscripts of different letters within each column indicate significant differences (*p* < 0.05) between mean values, as determined by Duncan’s test. CHC: commercial house cricket; PPF: house cricket fed with pure plant feed; 5% BSFL: the group fed substrate containing 5% BSFL powder; 10% BSFL: the group fed substrate containing 10% BSFL powder; SFA: saturated fatty acid; MUFAs, monounsaturated fatty acids; PUFAs: polyunsaturated fatty acids; AI: atherogenicity index; TI: thrombogenicity index.

## Data Availability

The original contributions presented in this study are included in the article/Supplementary Materials. Further inquiries can be directed to the corresponding authors.

## Supplementary Materials

The following supporting information can be downloaded at: https://www.mdpi.com/article/10.3390/foods14071140/s1, Table S1: Osborne solubility protein fractions of different *A. domesticus*; Table S2: The proportion of Osborne soluble protein components in different *A. domestic* powders; Table S3: The AAS, SC, and CS of protein in different *A. domestic* powder; Table S4: Digestibility and hydrolysis degree of protein in *A. domesticus* reared on different feeds during in vitro digestion; Table S5: The antioxidant activity and enzyme inhibitory activities of protein in different *A. domesticus* during in vitro digestion.
