# Supplementary material for: The Addition of Hermetia illucens to Feed: Influence on Nutritional Composition, Protein Digestion Characteristics, and Antioxidant Activity of Acheta domesticus
Source: Foods. 2025 Mar 25;14(7):1140. doi: 10.3390/foods14071140 (PMC11988907; doi:10.3390/foods14071140)
Supplement: Supplementary file 1 [file foods-14-01140-s001.zip › foods-3534381-supplementary.pdf]

Table S1

Osborne solubility protein fractions of different *A. domesticus*.

	CHC	PPF	5%-BSFL	10%-BSFL	*p*-value
Albumin	3.38 ± 0.38^{a}	11.46 ± 0.31^{c}	8.80 ± 0.38^{b}	8.21 ± 0.34^{b}	< 0.001
Globulin	4.60 ± 0.28^{a}	18.69 ± 0.42^{d}	11.67 ± 0.80^{b}	17.19 ± 0.80^{c}	< 0.001
Prolamin	5.62 ± 0.25^{a}	5.63 ± 0.27^{a}	7.34 ± 0.41^{b}	5.61 ± 0.16^{a}	< 0.001
Glutelin	141.06 ± 0.38^{a}	199.06 ± 0.31^{c}	218.72 ± 0.38^{d}	194.08 ± 0.34^{b}	< 0.001
Total	154.66 ± 3.66^{a}	234.84 ± 2.34^{c}	246.54 ± 4.62^{d}	225.09 ± 3.90^{b}	< 0.001

CHC: commercial house cricket; PPF: house cricket fed with pure plant feed; 5% BSFL: the group fed substrate containing 5% BSFL powder; 10% BSFL: the group fed substrate containing 10% BSFL powder. Superscripts of different letters within each column indicate significant differences ($p < 0.05$) between mean values, as determined by Duncan's test.

Table S2

The proportion of osborn solublity protein components in different *A. domestic* powder.

	CHC	PPF	5%-BSFL	10%-BSFL	*p*-value
Albumin	2.18 ± 0.19^{a}	4.88 ± 0.18^{c}	3.57 ± 0.09^{b}	3.65 ± 0.09^{b}	< 0.001
Globulin	2.97 ± 0.11^{a}	7.96 ± 0.10^{c}	4.73 ± 0.23^{c}	7.64 ± 0.23^{b}	< 0.001
Prolamin	3.63 ± 0.08^{c}	2.40 ± 0.09^{a}	2.98 ± 0.11^{b}	2.49 ± 0.04^{a}	< 0.001
Glutelin	91.21 ± 0.38^{d}	84.76 ± 0.02^{a}	88.72 ± 0.43^{c}	86.23 ± 0.34^{b}	< 0.001

CHC: commercial house cricket; PPF: house cricket fed with pure plant feed; 5% BSFL: the group

fed substrate containing 5% BSFL powder; 10% BSFL: the group fed substrate containing 10% BSFL powder. Superscripts of different letters within each column indicate significant differences ($p < 0.05$) between mean values, as determined by Duncan's test.

Table S3

The AAS, SC and CS of protein in different *A. domestic* powder.

	CHC	PPF	5%-BSFL	10%-BSFL	*p*-value
			AAS		
His	1.72 ± 0.00	1.68 ± 0.11	1.63 ± 0.02	1.70 ± 0.07	0.381
Thr	1.19 ± 0.02^{a}	1.21 ± 0.04^{a}	1.22 ± 0.00^{a}	1.28 ± 0.03^{b}	0.039
Val	1.06 ± 0.00^{a}	1.11 ± 0.07^{a}	1.20 ± 0.02^{b}	1.19 ± 0.03^{b}	0.007
Leu	1.02 ± 0.01^{a}	1.06 ± 0.05^{a}	1.01 ± 0.02^{ab}	1.11 ± 0.02^{b}	0.012
Ile	0.99 ± 0.02^{a}	1.08 ± 0.05^{b}	1.10 ± 0.04^{bc}	1.17 ± 0.02^{c}	0.003
Lys	0.83 ± 0.01	0.83 ± 0.05	0.85 ± 0.02	0.86 ± 0.01	0.563
Phe+Tyr	1.38 ± 0.03^{a}	1.42 ± 0.09^{ab}	1.65 ± 0.03^{b}	1.51 ± 0.05^{c}	0.001
Met+Cys	0.68 ± 0.05	0.68 ± 0.08	0.73 ± 0.00	0.72 ± 0.10	0.74
Limiting amino acid	Met+Cys	Met+Cys	Met+Cys	Met+Cys	
			RC		
His	1.55 ± 0.01^{b}	1.48 ± 0.09^{ab}	1.39 ± 0.01^{a}	1.43 ± 0.06^{a}	0.031
Thr	1.07 ± 0.02	1.07 ± 0.02	1.04 ± 0.02	1.07 ± 0.01	0.118
Val	0.96 ± 0.01^{a}	0.98 ± 0.01^{b}	1.02 ± 0.01^{bc}	1.00 ± 0.02^{c}	0.001
Leu	0.92 ± 0.02^{b}	0.94 ± 0.00^{b}	0.86 ± 0.00^{a}	0.93 ± 0.01^{b}	< 0.001

Ile	0.89 ± 0.02^{a}	0.95 ± 0.02^{bc}	0.94 ± 0.02^{b}	0.98 ± 0.02^{c}	0.003
Lys	0.75 ± 0.01	0.73 ± 0.02	0.72 ± 0.01	0.72 ± 0.01	0.074
Phe+Tyr	1.24 ± 0.02^{a}	1.25 ± 0.02^{a}	1.41 ± 0.01^{b}	1.26 ± 0.03^{a}	< 0.001
Met+Cys	0.61 ± 0.04	0.60 ± 0.06	0.62 ± 0.01	0.60 ± 0.09	0.973
Limiting amino acid	Met+Cys	Met+Cys	Met+Cys	Met+Cys	
CS					
His	1.22 ± 0.00	1.19 ± 0.08	1.15 ± 0.02	1.20 ± 0.05	0.372
Thr	0.62 ± 0.01^{a}	0.63 ± 0.02^{a}	0.64 ± 0.00^{ab}	0.67 ± 0.01^{b}	0.021
Val	0.62 ± 0.00^{a}	0.65 ± 0.04^{a}	0.70 ± 0.01^{b}	0.70 ± 0.02^{b}	0.003
Leu	0.64 ± 0.01^{a}	0.67 ± 0.03^{ab}	0.64 ± 0.01^{a}	0.70 ± 0.01^{b}	0.021
Ile	0.52 ± 0.01^{a}	0.56 ± 0.03^{b}	0.57 ± 0.02^{bc}	0.61 ± 0.01^{c}	0.004
Lys	0.57 ± 0.01	0.57 ± 0.04	0.58 ± 0.02	0.59 ± 0.01	0.39
Phe	0.41 ± 0.01^{a}	0.43 ± 0.03^{ab}	0.44 ± 0.01^{ab}	0.47 ± 0.02^{b}	0.048
Met	0.30 ± 0.04	0.31 ± 0.02	0.34 ± 0.00	0.31 ± 0.08	0.73
Limiting amino acid	Met	Met	Met	Met	

CHC: commercial house cricket; PPF: house cricket fed with pure plant feed; 5% BSFL: the group fed substrate containing 5% BSFL powder; 10% BSFL: the group fed substrate containing 10% BSFL powder, AAS: amino acid score, RC: acid ratio coefficient; CS: chemical score. Superscripts of different letters within each column indicate significant differences ($p < 0.05$) between mean values, as determined by Duncan's test.

Table S4

Digestibility and hydrolysis degree of protein in *A. domesticu* reared on the different feed during *in vitro* digestion.

	CHC	PPF	5%-BSFL	10%-BSFL	*p*-value
Protein digestibility (%)					
0	6.31 ± 1.52^{a}	13.13 ± 2.50^{b}	11.82 ± 2.38^{b}	10.66 ± 1.82^{b}	0.019
30	16.07 ± 1.69^{a}	20.27 ± 2.37^{b}	29.01 ± 1.41^{c}	23.46 ± 2.03^{b}	< 0.001
60	19.15 ± 1.83^{a}	25.25 ± 0.94^{b}	32.58 ± 3.06^{c}	30.47 ± 3.31^{c}	0.001
90	21.07 ± 3.35^{a}	28.12 ± 1.41^{b}	34.85 ± 1.24^{c}	35.65 ± 0.48^{c}	< 0.001
120	31.06 ± 1.21^{a}	33.41 ± 4.26^{ab}	36.37 ± 2.29^{bc}	38.59 ± 1.00^{c}	0.03
Degree of hydrolysis (%)					
0	4.22 ± 0.07^{a}	4.91 ± 0.06^{b}	5.31 ± 0.03^{c}	4.82 ± 0.03^{b}	< 0.001
30	7.58 ± 0.33^{a}	8.82 ± 0.48^{b}	9.71 ± 0.23^{c}	9.26 ± 0.27^{bc}	< 0.001
60	9.12 ± 0.38^{a}	10.82 ± 0.41^{b}	11.82 ± 0.07^{c}	11.37 ± 0.26^{bc}	< 0.001
90	10.61 ± 0.22^{a}	11.77 ± 0.21^{b}	12.56 ± 0.33^{c}	12.54 ± 0.33^{c}	< 0.001
120	11.14 ± 0.57^{a}	12.71 ± 0.69^{b}	13.38 ± 0.51^{b}	12.93 ± 0.31^{b}	0.004

CHC: commercial house cricket; PPF: house cricket fed with pure plant feed; 5% BSFL: the group fed substrate containing 5% BSFL powder; 10% BSFL: the group fed substrate containing 10% BSFL powder. Superscripts of different letters within each column indicate significant differences ($p < 0.05$) between mean values, as determined by Duncan's test.

Table S5

The antioxidant activity and enzyme inhibitory activities of protein in different *A. domesticu* during in *vitro* digestion.

	CHC	PPF	5%-BSFL	10%-BSFL	*p*-value
DPPH	1.23 ± 0.09^a	1.37 ± 0.06^b	1.20 ± 0.08^a	1.17 ± 0.06^a	0.049
ABTS	1.03 ± 0.01^c	1.00 ± 0.02^b	0.76 ± 0.00^a	0.77 ± 0.00^a	< 0.001
OH-	1.72 ± 0.01^b	1.52 ± 0.08^a	1.49 ± 0.06^a	1.46 ± 0.01^a	0.001
TRP	2.35 ± 0.08^c	2.28 ± 0.06^c	2.13 ± 0.06^b	1.95 ± 0.05^a	< 0.001
FRAP	0.94 ± 0.01^b	0.98 ± 0.04^b	0.85 ± 0.02^a	0.81 ± 0.05^a	< 0.001
CUPRAC	0.83 ± 0.04^b	0.84 ± 0.03^b	0.73 ± 0.03^a	0.71 ± 0.03^a	0.002

CHC: commercial house cricket; PPF: house cricket fed with pure plant feed; 5% BSFL: the group fed substrate containing 5% BSFL powder; 10% BSFL: the group fed substrate containing 10% BSFL powder, DPPH: DPPH radical scavenging activity, ABTS: ABTS radical scavenging activity, OH-: hydroxide radical (OH-) scavenging activity, TRP: potassium hexacyanoferrate (III) total reducing power assay, FRAP: ferric reducing antioxidant power, CUPRAC: cupric ion reducing activity (CUPRAC). Superscripts of different letters within each column indicate significant differences ($p < 0.05$) between mean values, as determined by Duncan's test.
